# Left ventricular torsion: feeling the heat

**DOI:** 10.1113/expphysiol.2010.055806

**Published:** 2011-02

**Authors:** Rory B Weiner, Aaron L Baggish

During systole, the base and apex of the left ventricle (LV) rotate along the longitudinal axis of the LV in opposite directions. When viewed from an apical reference point, systolic rotation at the apex is anticlockwise while the base rotates in an overall clockwise direction. This counter-directional rotation results in a wringing motion of the heart, known as LV torsion (LVT) or twist. The terms ‘torsion’ and ‘twist’ refer to the same phenomenon and define the base-to-apex gradient in rotation. In addition to the systolic phenomenon of LVT, subsequent recoil and untwisting during early diastole is an important component of LV filling. This is commonly measured as peak early diastolic untwisting rate (UTR). The term ‘LV twist mechanics’ encompasses both systolic LVT and diastolic UTR and is an important contributor to overall LV function. Despite recent advances in the understanding of LV twist mechanics, determinants of the magnitude of LVT and UTR remain incompletely characterized.

Both measurement technique and physiological factors are determinants of observed LVT. Variability attributable to measurement technique may arise from imprecise standardization of the level of apical and basal image acquisition, sampling of different regions of the myocardium (i.e. subendocardium *versus* subepicardium), and failure to account for potentially important differences in LV geometry (i.e. normalization for left ventricular length). Physiological determinants of LVT include but are not limited to cardiac loading conditions, myocardial contractility, structural heart disease, heart rate, age and exercise training. Previous animal work has investigated the role of several of these factors, and our group (Weiner *et al.* 2010*a*) as well as others (Nelson *et al.* 2010) have studied these factors in humans.

In this issue of *Experimental Physiology*, Stöhr *et al.* (2011) report a thoughtfully designed and conducted study of LV twist mechanics during the following four conditions: (1) rest in normothermia; (2) exercise in normothermia; (3) rest with progressive hyperthermia; and (4) exercise with progressive hyperthermia. The main findings were that LVT and UTR increased significantly with progressive heat stress during resting conditions. Importantly, these changes in LV twist mechanics were accompanied by an increase in cardiac output and reductions in both LV end-diastolic and end-systolic volumes. In a similar fashion, increases in LVT and UTR were observed during normothermic exercise. However, the addition of heat stress to exercise was only associated with a non-significant trend towards further augmentation of LVT and UTR. This appears to be due to an increase in LVT/UTR with mild heat stress and then a plateau or decrease with additional levels of heat stress.

The authors are to be commended for conducting a physiological study to address the hypothesis that augmentation of LV twist mechanics helps maintain cardiac function in the face of heat stress. Several of their findings merit further discussion. First, it is noteworthy that the increased LVT seen with heat stress at rest was secondary to an increase in basal rotation (with no significant change in the magnitude of apical rotation). This is in contrast to the majority of previous studies evaluating LVT before and after a haemodynamic perturbation, in which differences in LVT were driven by changes in apical rotation (with relatively stable basal rotation). The authors of the present study speculate that the associated reduction in preload occurring with heat stress may limit changes in LV apical rotation. Although this observation deserves further study, previous work has demonstrated that apical rotation is highly preload dependent (Weiner *et al.* 2010*b*).

Second, the finding of an ‘uncoupling’ of LVT and LV strain is intriguing. As described earlier, LVT results from the counter-directional rotation of the LV base and apex. Left ventricular strain is a dimensionless measurement of deformation, expressed as a fractional or percentage change from an object's original dimension. It can be measured in several vectors, including longitudinal, circumferential and radial. Both LV strain and LVT are validated measures of systolic function, and conventionally these parameters are thought to change in a concordant fashion. However, Stöhr *et al.* (2011) report an increase in LVT (and LV ejection fraction), although there were no significant changes in LV strains. In a similar fashion, another recent report of LVT during progressive submaximal exercise also showed that LVT increased progressively with exercise intensity while longitudinal strain remained unchanged (Doucende *et al.* 2010). This raises the possibility that LVT reserve is important for maintaining cardiac function during haemodynamic challenge independent of LV strain. However, as the present study suggests, there may be a limit to this reserve, because the combined challenge of heat stress and exercise did not lead to a further increases in LVT. Clarifying the relative contributions of LVT and LV strain during physiological challenges represents an area of future work.

Third, the principal finding of this paper is the differential response of LV twist mechanics during rest *versus* exercise heat stress. The mechanisms underlying this are complex and probably reflect the multitude of factors contributing to the magnitude of LVT and UTR. The observation that mean arterial pressure (LV after-load) decreased in the exercise heat stress group (and not in the rest heat stress group) is offered as a potential explanation for the different LV twist mechanics responses. This observed decline in mean arterial pressure during hyperthermic isotonic exercise indicates a significant change in cardiac loading conditions. The interplay between loading conditions and LV twist mechanics, which contributes to the findings of the present study, is not completely understood. Additionally, the finding of a lack of further increase in LV twist mechanics with high levels of heat stress and exercise may suggest that there is a physiological limit to LVT/UTR augmentation.

Novel studies, such as the present investigation by Stöhr *et al.* (2011), provide insight into our understanding of cardiac function in response to physiological perturbations. Further study of LV twist mechanics with refined speckle tracking echocardiographic techniques has the potential to better elucidate myocardial systolic and diastolic adaptations to physiological stressors. Ultimately, this will help provide a framework for better understanding of the role of LV twist mechanics in human performance and cardiac disease.
